# Assessing and monitoring the impact of the national newborn hearing screening program in Israel

**DOI:** 10.1186/s13584-019-0296-6

**Published:** 2019-03-11

**Authors:** Janice Wasser, Daphne Ari-Even Roth, Orly Herzberg, Liat Lerner-Geva, Lisa Rubin

**Affiliations:** 1 Department of Maternal and Child Health, Public Health Services, Ministry of Health, Jerusalem, Israel; 2 Department of Communication Disorders, School of Health Professions, Sackler Faculty of Medicine, Tel Aviv University, Tel Aviv, Israel; 3 Hearing, Speech and Language Center, Sheba Medical Centre, Tel HaShomer, Israel; 4 Chief Communication Disorders Clinician, Medical Directorate, Ministry of Health, Tel Aviv, Israel; 5 Women and Children’s Health Research Unit, The Gertner Institute for Epidemiology and Health Policy, Tel HaShomer, Israel; 6 School of Public Health, Sackler Faculty of Medicine, Tel Aviv University, Tel Aviv, Israel; 7 School of Public Health, University of Haifa, Haifa, Israel

**Keywords:** Newborn hearing screening, Congenital hearing loss, Early intervention, National public health program, Compliance

## Abstract

**Background:**

The Israeli Newborn Hearing Screening Program (NHSP) began operating nationally in January 2010. The program includes the Otoacoustic Emissions (OAE) test for all newborns and Automated Auditory Brainstem Response (A-ABR) test for failed OAE and infants at risk for auditory neuropathy spectrum disorders. NHSP targets are diagnosis of hearing impairment by age three months and initiation of habilitation by six months.

**Objectives:**

(1) Review NHSP coverage; (2) Assess NHSP impact on age at diagnosis for hearing impairment and age at initiation of habilitation; (3) Identify contributing factors and barriers to NHSP success.

**Methods:**

(1) Analysis of screening coverage and referral rates for the NHSP; (2) Analysis of demographic data, results of coverage, age at diagnosis and initiation of habilitation for hearing impaired infants pre-implementation and post-implementation of NHSP from 10 habilitation centers; (3) Telephone interviews with parents whose infants failed the screening and were referred for further testing.

**Results:**

The NHSP coverage was 98.7% (95.1 to 100%) for approximately 179,000 live births per year for 2014–2016 and average referral rates were under 3%. After three years of program implementation, median age at diagnosis was 3.7 months compared to 9.5 months prior to NHSP. The median age at initiation of habilitation after three years of NHSP was 9.4 months compared to 19.0 prior to NHSP. Parents (84% of 483 sampled) with infants aged 4–6 months participated in the telephone survey. While 84% of parents reported receiving a verbal explanation of the screening results, more than half of the parents reported not receiving written material. Parental report of understanding the test results and a heightened level of concern over the failed screen were associated with timely follow-up.

**Conclusions:**

The findings indicate high screening coverage. The program reduced ages at diagnosis and initiation of habilitation for hearing impaired infants. Further steps needed to streamline the NHSP are improving communication among caregivers to parents to reduce anxiety; increasing efficiency in transferring information between service providers using advanced technology while ensuring continuum of care; reducing wait time for follow-up testing in order to meet program objectives. Establishment of a routine monitoring system is underway.

## Background

The importance of early identification of hearing impairment is well established. Accumulating evidence shows that undiagnosed or untreated permanent hearing impairment (PHI) during early childhood may result in speech-language delay, poor academic achievements, and social-emotional difficulties [1]. Such delays in the different domains have been documented also for those whose PHI was mild-to-moderate or only in one ear [2–4]. Currently, there is overwhelming evidence showing that early diagnosis and habilitation before the age of six months improves speech and language development and cognitive outcomes, decreasing the need for special education and improving quality of life [5–7].

In order to identify hearing-impaired infants as early as possible and offer them the appropriate intervention, the National Institute of Health (NIH) in 1993 recommended the implementation of universal neonatal hearing screening programs (NHSP) up to the age of three months in order to initiate hearing habilitation no later than the age of six months [8]. The Israeli national hearing screening program at that time was conducted at Mother and Child Health Clinics (MCHC) using the distraction test at age 7–9 months. Since 1997, a number of medical centers in Israel began offering NHSP. The Ministry of Health (MOH) Directive 33/2009 established the guidelines for the NHSP for all infants to be implemented from January 1st, 2010 [9]. Following the recommendations stated in these guidelines, the current program consisting of the Otoacoustic Emissions (OAE) test for all newborns and Automated Auditory Brainstem Response (A-ABR) test for those infants who failed OAE testing and for infants at risk for auditory neuropathy spectrum disorders (ANSD) was established. The national program objectives are to complete screening by age one month, conduct diagnostic audiologic testing no later than age three months for those infants who failed the screening and initiate habilitation for those diagnosed with hearing loss by age six months. Early screening covers all newborns in the country and continues for those who failed the secondary screens and those identified as high-risk groups.

With the NHSP running for several years, we aimed to assess and monitor the progress of the Israeli program. Specifically, the goals of the present study were to evaluate: (1) screening coverage and referral rates for the NHSP; (2) the impact of NHSP on the age at diagnosis and age at initiation of habilitation comparing pre- and post-implementation; (3) parental satisfaction and compliance with follow up; (4) the factors which support or impede the use of services for infants who failed the newborn hearing screen.

## Methods

**Fig. 1:**
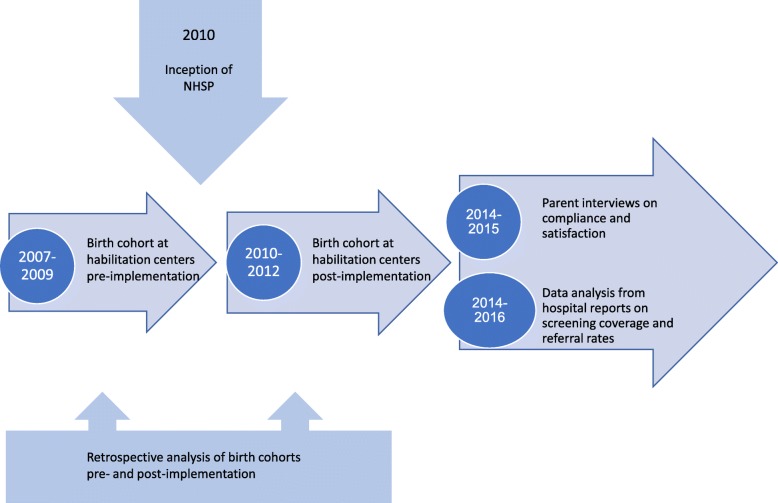
Timeline of study parameters

The study parameters for the multi-tiered research are described in Fig. 1. Data presented in this study were measured from three different sources and time periods. The first source was the chart review from the 10 Habilitation Centers for the pre- and post-implementation of the NHSP (2007–2010) analyzing the age at diagnosis and age at initiation to habilitation. The second source was the telephone survey measuring parental compliance and satisfaction with the NHSP (2014–2015) and the third source was hospital reported data for analyzing coverage of screening and referral rates for diagnostic screening (2014–2016).

This design of the present study was based on the model provided by the New South Wales Department of Health on its Statewide Infant Screening Hearing evaluation (NSW SWISH) Program published in May 2011 [10]. The objectives for that evaluation were to assess the appropriateness, effectiveness and efficiency of the program in meeting the performance indicators and international benchmarks for newborn hearing screening programs; to evaluate processes including: program management, referral processes, service networks and the satisfaction of parents/families; and to develop a monitoring system and make recommendations for ongoing management and reporting of all aspects of the program.

This comprehensive design for evaluation as well as the similarity in the division of responsibility for screening and follow up that exists in Israel and in New South Wales offer an appropriate model for comparison and has been most helpful in determining the strengths and weaknesses of the Israeli program and which steps should be taken in order to improve the services offered to families in Israel.

### Coverage of screening and referral rates

NHSP coordinators based in the hospitals are required to provide an annual report to the Mother and Child Health Department of the Ministry of Health on the screening program since 2012. In actuality, the reports were only sufficiently complete from 2014 onwards. The testing protocol consists of up to two OAE screens (DP is used in nine hospitals, TE in 17 hospitals) and an A-ABR screen for a failed OAE. The protocol for infants at high risk for ANSD consists of both OAE and A-ABR screens.

Data included number of live births, infant deaths or transfers to other hospitals, infants completing the full protocol indicated, OAE results, A-ABR results for failed OAE, number of high risk (HR) infants with failed A-ABR and referral rates. Aggregate data is reported based on each hospital’s method of recording screening results. Coverage rates were computed from the aggregate data to produce a summary report at the end of each year. The latest report is dated October, 2017.

Referral rates (RR) are calculated by taking the number of infants who failed their neonatal screen and referred to diagnostic audiologic testing divided by the total number of infants screened in the NHSP by hospital.

### Age at diagnosis, age at initiation of habilitation

Field research was conducted in all 10 habilitation centers and their satellites offering services for children with hearing disorders. After approval from the Ministry of Health’s Ethics Committee (No. 067/2011) was obtained, the files for children attending the centers born during 2007–2009 (pre-NHSP implementation birth cohort) (*n* = 431), and during 2010–2012 (post-NHSP implementation birth cohort) (*n* = 428) were reviewed. Data was collected from the child’s chart on demographics, birth, date of diagnosis, date of initiation of habilitation and severity of the hearing loss recorded using a structured questionnaire. The age at initial diagnosis was defined as the chronological age of the child on the date of the first diagnostic audiologic testing after a positive screening. The age of final diagnosis was calculated as the chronological age of the child for the last diagnostic testing recorded before the child began habilitation. No age correction was applied for preterm infants (*n* = 67 for pre-implementation cohort, *n* = 80 for post-implementation cohort) since no change was observed in the outcomes when they were removed from the analysis. Three categories for severity of hearing loss were used: 26–40 dB mild, 41–70 dB moderate and > 70 severe to profound. Information regarding measured severity of hearing loss was available for 96% (*n* = 824) of the children.

The data extracted from the habilitation center charts were for two time periods, before and after initiation of national screening and focused on the two parameters of age at initial diagnosis and age at initiation to habilitation in order to measure the impact of the NHSP post-implementation. The habilitation centers treat children with both congenital and later onset hearing loss. However, information regarding the etiology of their hearing disorders or the level of severity over time was not available for all children. Since children with hearing loss subsequent to meningitis were easily identified and not targets of the neonatal hearing screen, they were excluded from the study. While the outcome measures of age at diagnosis and age at initiation of habilitation included both children with congenital and those with late onset or progressive hearing loss, assessing the distribution of these measures and their changes subsequent to implementation of neonatal screening is one method of assessing impact of the program.

### Compliance and parent satisfaction

Approval from the Ministry of Health’s Ethics Committee (No. 16/2015) was obtained in order to access computerized clinical health records of all government run MCHCs and to conduct a telephone survey to measure compliance and parent satisfaction with the NHSP.

Approximately 70% of Israeli infants receive preventive care in the government run MCHCs. Of the children enrolled, 98% had a screen result documented in their record. The sampling frame included infants 4–6 months of age (born between August 2014 and May 2015). Parents of these children who had a telephone number^1^ and agreed to participate in the survey were interviewed (*n* = 483). The telephone interview was scheduled to take place by six months after birth since repeat testing was expected to be completed by this age.

The distribution of the socio-economic status (SES) by place of residence for families surveyed was compared to the general population in which the division of clusters 1–3, 4–6 and 7–9 (one being the lowest SES, nine being the highest) used by the Israeli Central Bureau of Statistics are approximately represented equally; 34, 31 and 33%. In our sample, there was a higher representation of the mid to lower SES (35, 49, 15%) which is expected since this population is served by the government run MCHCs.

The study sample consisted of fairly equal numbers of the two main population groups in Israel, Jews and Arabs. The Arab population (21% of the total population of Israel [11]) is over-represented in this sample as we were especially interested in examining possible gaps in the access to services for this population. The sample population was 36% female, 48% Arab and the average age of the infant at time of the interview was 6.3 months.

The telephone interview was scheduled to take place by six months after birth since repeat hearing testing was expected to be completed by this age. The interview was conducted with one of the parents using an electronically-generated structured questionnaire about their experience with the neonatal hearing screening test through to follow up for further testing after discharge (*n* = 405).

The questionnaire covered four main aspects of the screening process for newborns: how parents were informed of test results, anxiety level following positive results, compliance with referral for follow up testing, final diagnosis, and satisfaction with the screening process. The telephone interviews were conducted in Hebrew and Arabic.

Statistical analyses were performed using Z scores and T tests to compare the characteristics of the pre- and post-implementation groups and measured outcomes. Chi square tests were used to determine whether there was an association between the time to follow up testing and the factors measured in the telephone survey.

## Results

### Coverage of screening and referral rates for diagnostic testing

Routine regulatory visits by Ministry of Health staff have found that NHSP was routinely performed in all 28 birthing hospitals across the country (OH). Full aggregate reports were available for 26 of the 28 hospitals in 2016. Three hospitals reported having partial or complete absence of computerized reporting software for screening results. The following report refers to the analyzable data for 2014 to 2016. In 2015, the year with fewest hospitals reporting, over 95% of the births in Israel were included. For the three years studied, the average number of births was just under 179,000 per year [12].

Reporting on coverage of the full protocol was 98.3% in 2014 (25 hospitals), 99.2% in 2015 (24 hospitals) and 98.7% in 2016 (26 hospitals). In 2016, the range was from 95.1 to 100% for 26 hospitals, with only four hospitals reporting coverage under 98%. The two hospitals reporting the lowest coverage (86.0 and 95.7%) in 2014 were able to increase their coverage to 99.0 and 98.9% in 2016, yet three hospitals showed a reduction in coverage from 100, 99.9 and 99.0% in 2014 to 99.7, 97.0 and 95.1% in 2016. Two hospitals, which hadn’t provided full reports in 2014 and 2015, reported coverage of 100 and 99.0% in 2016.

In the majority of hospitals, the rate of reporting for full coverage of the protocol and OAE alone were almost identical. One hospital reported that OAE testing coverage was significantly higher than the full protocol coverage, suggesting difficulty in conducting the subsequent A-ABR tests.

The average RR was 1.8% in 2014, 2.5% in 2015 and 2.6% in 2016. Twenty of the hospitals had a referral rate of less than the 4% benchmark [13]. Of the six hospitals reporting a RR equal to or greater than 4% in 2016, four showed an increase from 2014 whereas two had RR’s that were consistently high. The number of hospitals reporting RR under 1% declined over the years, from 10 in 2014 to five in 2016. Only one hospital consistently reported a RR of less than 1%. No association was found between referral rates and the annual number of births per hospital.

### Age at initial diagnosis, age at initiation of habilitation

The demographic and obstetric characteristics of the “pre-NHSP implementation” and “post-NHSP implementation” cohorts are presented in Table 1. The groups were comparable with the exception of a significantly greater proportion of Arabs in the post-NHSP implementation cohort.

**Table 1:** Comparison of the “pre-NHSP implementation” and “post-NHSP implementation” cohorts by demographic and obstetric characteristics

Characteristics of samples	2007-2009 *N*= 431^a^	2010-2012 *N*=428^a^	Z scores & T tests
Female	47%	46.2%	*p* = .841
Jews and others^b^	77.7%	66.8%	***p*** **= 0.0002**
Arabs	22.3%	33%	***p*** **< 0.0005**
Mean birthweight (grams)	2964	2936	*p* = .627
Mean gestational age (weeks)	38.2	38.0	*p* = .334
Extremely preterm (< 28 weeks)	2.2%	2.5%	*p* = .578
Moderately preterm (28-36 weeks)	14.5%	17%	*p* = .679
Severity of hearing disorder			
Unilateral	*n* = 54	*n* = 21	
Mild	1%	0	
Moderate	6%	2%	
Profound	6%	3%	
Bilateral	*n* = 343	*n* = 243	
Mild	7%	10%	
Moderate	29%	28%	
Profound	43%	50%	

^a^missing data are not included in calculations

^b^others are non-Arab minorities and cases with “unknown” population group

**Fig. 2:**
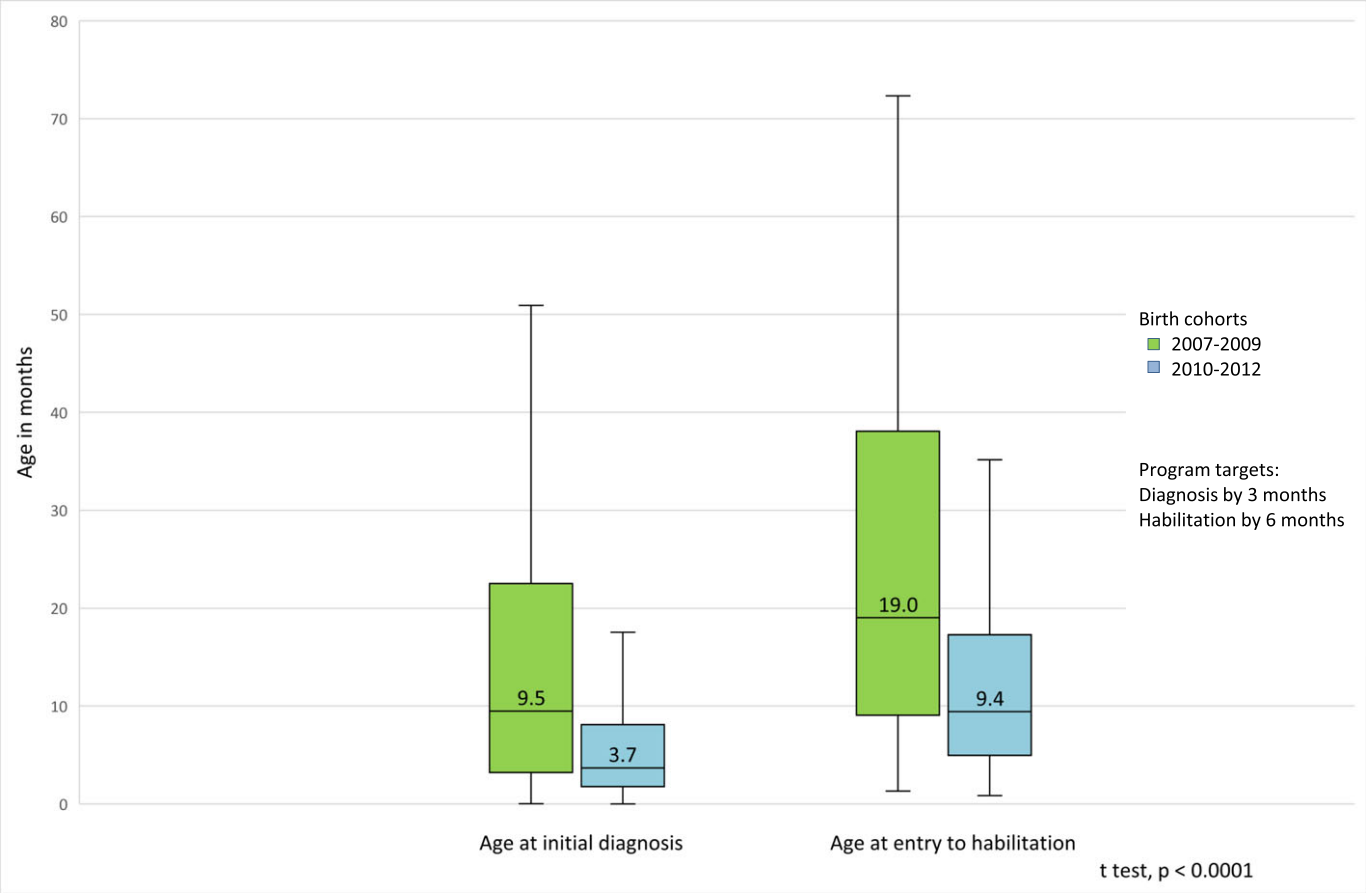
Age at initial diagnosis and age at initiation of habilitation, pre- and post-implementation of the Israeli NHSP by birth cohort

The median age at initial diagnosis decreased by 5.8 months, from 9.5 to 3.7 months for the first three years following implementation. The median age at initiation of habilitation was reduced by 9.6 months, from 19.0 to 9.4 months (Fig. 2). Despite the marked improvement following implementation, 50% of infants born from 2010 to 2012 were still diagnosed after age three months and 64.5% were enrolled in habilitation centers after age six months.

**Fig. 3:**
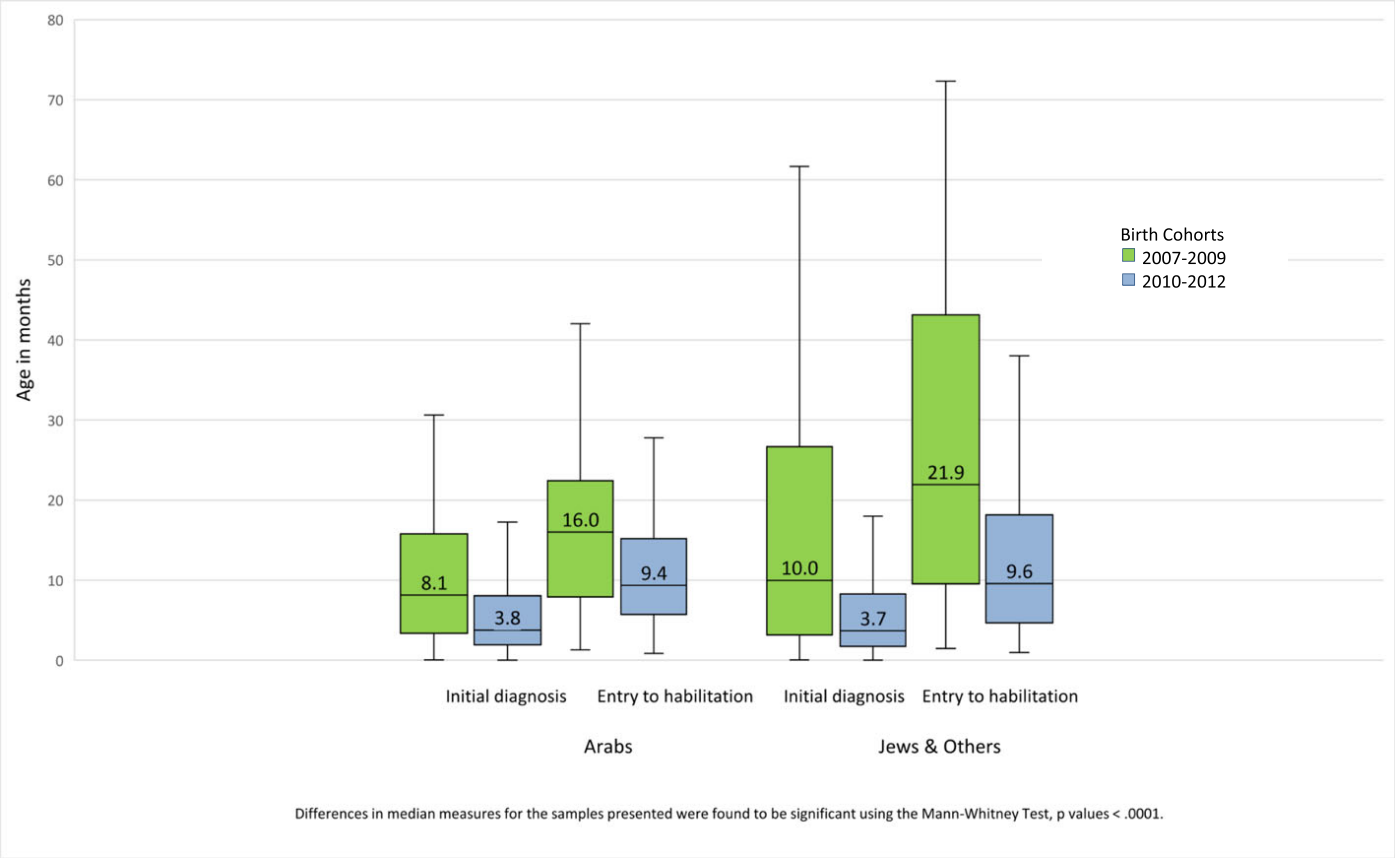
Age at initial diagnosis and age at initiation of habilitation, pre- and post-implementation of the Israeli NHSP by population group

The reduction after implementation in both median age at initial diagnosis and median age at initiation of habilitation were greater for Jews and others than for the Arab population (see Fig. 3). The median age of initial diagnosis and initiation of habilitation before implementation started out higher for the Jewish population than for the Arab population studied, yet the post implementation ages for all children were approximately the same.

### Parental compliance and satisfaction

**Fig. 4:**
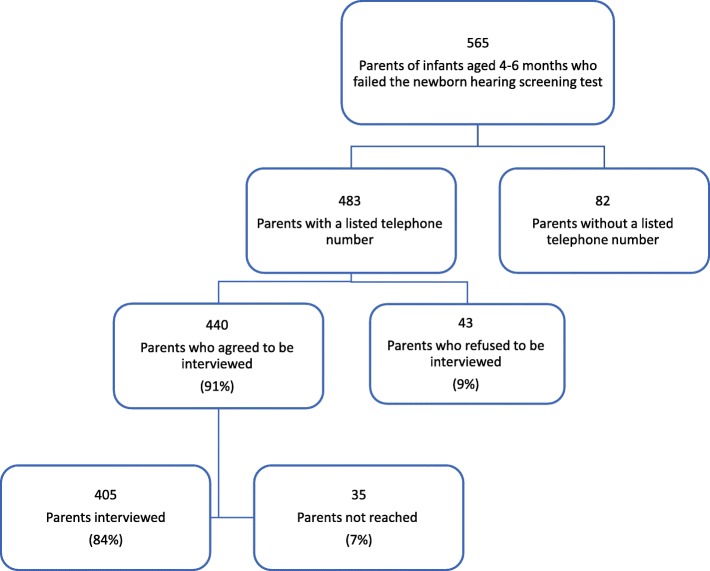
Description of sample for telephone interview on parental satisfaction and compliance with NHSP for 2014–2015

In order to examine the factors affecting the age at diagnosis and initiation of habilitation we surveyed 405 parents of infants with a failed hearing screen regarding compliance and satisfaction with the NHSP. The sample was drawn from records of 483 infants with a report of a failed screen entered into the MCHC well-child health database. The report of a failed screen was based on the written hospital discharge summary for 98% of the infants. Response rate was 84% (*n* = 405). Description of the sample is illustrated in Fig. 4. The study sample consisted of fairly equal numbers of the two main population groups in Israel, Jews and Arabs. The Arab population (21% of the total population of Israel [11]) is over-represented in this sample as we were especially interested in examining possible gaps in the access to services for this population. The sample population was 36% female, 48% Arab and the average age of the infant at time of the interview was 6.3 months.

Of the 405 families interviewed, 394 parents reported on their experience of the infant hearing screen conducted in the hospital after birth. Three families were unable to answer questions regarding the screening experience because of the poor health status of their child at birth, and one family said they were released early because of the Sabbath. Data were missing for seven families. Discrepancies were detected for 45 families who reported that their child passed the screen despite hospital records indicating a failed screen. Seven infants were described as high risk.

Overall 83% (*n* = 326) of parents of infants with a documented failed screen reported having performed follow up testing of which 81% (*n* = 265) were seen within three months after discharge from the hospital. Of those examined within three months, 27 infants (10%) failed the repeat test. For those seen more than three months after discharge (*n* = 54), four infants failed the test. There were 47 infants without final results at the time of the interview. Two families declined follow up testing. There were seven infants (2%) whose parents reported not being referred in spite of a failed screen.

Table 2 provides data collected from the parental interviews to measure whether certain factors were associated with compliance by time to follow up testing. Appropriate follow up was designated as a test up to three months after discharge from the hospital, delayed follow up was a test more than three months after discharge, and no follow up was no report of a referral or follow up test. Probability of the outcomes was assessed using chi-square tests exhibiting the *p* values indicated. Parental report of understanding the test results and a heightened level of concern over the failed screen were found to be associated with timely follow-up. One third of the parents reported they were very concerned about the results of the neonatal hearing screen. While 84% of all those interviewed reported receiving a verbal explanation of the test results, over 50% of parents said they did not receive written explanatory materials.

**Table 2:** Factors affecting follow up testing^a^

Factor	% Follow up ≤ 3 mos	% Follow up > 3 mos	% No F/U	*p* value for Chi Square test
Sufficient time for explanation on screening (*n* = 345)	80%	77%	74%	*p* = 0.598
Screen results understood by parents (*n* = 353)	89%	77%	90%	***p*** **= 0.030**
Received results on day of testing (*n* = 350)	76%	68%	83%	*p* = 0.246
Heightened level of concern over screening result (*n* = 392)	55%	60%	29%	***p*** **= 0.001**
Hospital staff scheduled f/u appointment (*n* = 346)	85%	71%	NR	unable to perform test

^a^The *n* values for each factor differed because of missing data. Probability of these outcomes was assessed using chi-square tests exhibiting the *p* values above

Despite the above reported associations there were indications of possible confusion regarding the screen results where parents reported their infants passed the screen while also reporting results of follow up testing.

## Discussion

Decision makers and stakeholders use program evaluation and performance measurement as complementary approaches to assess and monitor program implementation. Effective program evaluation is a systematic way to improve and account for public health services by involving procedures that are useful, feasible, ethical, and accurate. These procedures measure whether the program serves the needs of the target population; whether the program objectives are realistic, pragmatic and diplomatic; whether program staff behave ethically and with appropriate regard for the welfare of those involved and those affected; and finally, whether the measures reveal and convey technically accurate information [14, 15].

Government programs are required to show accountability through performance measurement which include stating program objectives and corresponding outcomes. These measures track outcomes by data gathered to monitor program performance. Results-based management is premised on principles that emphasize the importance of stating clear program and policy objectives, measuring and reporting program and policy outcomes. Changes to improve program operations and efficiency and effectiveness are expected to be driven by evidence of how well programs are doing in relation to stated objectives [16].

This assessment of the Israel NHSP provides performance measures of screening coverage with referral rates and the impact on age at diagnosis and initiation of habilitation, and program evaluation by examining factors supporting or impeding follow up testing.

In Israel, implementation of the screening program was developed without concomitant reporting requirements. Hospitals were mandated to screen the infants and MCHC staff routinely review hearing screen results and monitor follow up, however responsibility for actual follow up and diagnostic testing resides with the insuring health funds. Organizational and budgetary constraints led to this disjunction between the responsibility for the screening program and its monitoring but were necessary to permit initiation of the program. However, this absence of a mandatory reporting system makes it difficult to study and assess compliance with follow up and achievement of the program objectives.

Overall screening coverage was consistently high. The coverage rate in Israel averaging 98.7% for 2014–2016, is in line with reports from the United Kingdom (UK) 98.9%, the United States (US) 97.9% and Australia 97% [8, 17, 18]. The high coverage rate is of note considering the protocol requiring the completion of up to three screens within the 48 h of routine postnatal hospitalization and frequent absence of testing on Saturdays because of Sabbath observance [19]. Countries such as the US and Australia have programs which do not necessarily include two stage testing [17, 20, 21].

The Expert Panel Recommendations on Newborn Hearing Screening expect that no more than 4% of newborns be referred for diagnostic audiological evaluation [22]. High OAE fail rates drive up the program’s rate of secondary A-ABR testing which burdens the screening program’s resources for staff and time. Higher rates of A-ABR testing fuel higher referral rates with concomitant risk of encumbering available diagnostic services and increasing wait times.

Referral rates of 4% or higher were recorded in the study period from 2014 to 2016 for nine hospitals whereby only two of these hospitals consistently reported high RR for each year. Three hospitals reported high RR for two of the three years, and four hospitals reported high RR for one of the years.

Officially sanctioned reductions of postpartum hospitalization stay in hospitals with crowding from large numbers of births may contribute to higher referral rates due to screening performed shortly after birth as well as shortened intervals between tests. However, this does not explain all high referral rates. There is a need for in-house assessment to explain high referral rates in these hospitals to improve this parameter. Issuing recommendations as to when rescreening is acceptable as well as specifying requisite intervals between screening tests could reduce the need for full diagnostic testing.

Since initial performance measures from the hospital reports indicated high coverage for screening, we chose to concentrate on evaluating the impact of the program by examining the age at diagnosis and age at initiation of habilitation for children receiving treatment in habilitation centers throughout the country. After three years of program implementation, the median age at diagnosis and age at initiation of habilitation were significantly reduced. These reductions were compared to those reported by other countries. In Australia, Ching et al. reports on a prospective population-based study of 451 children at age three years post-implementation. The median age at diagnosis was 2.1 months and enrolment in educational intervention occurred within five months of diagnosis with a median age of eight months [23]. For the UK, findings for the evaluation of the first phase of the population-based NHSP, median age at first assessment was five weeks and median age of enrolment in a habilitation program was 10 weeks compared to the situation before the introduction of screening, median age at identification of bilateral permanent hearing loss in the UK was 26 months [24]. The smaller reductions in median ages measured for the Israeli NHSP may possibly result from the fact that our present neonatal program replaced a national distraction screening program performed between 7 and 9 months.

Our findings of earlier age at diagnosis and treatment are corroborated by findings of earlier age for children receiving cochlear implants. The situation in Israel regarding age at implantation is indicated by the number of cochlear implants performed for children aged 0–5 years from 2005 to 2015. The median age decreased from 2.28 years to 1.75 years during this period [Z. Haklai, personal communication, July 18, 2016].

While not directly examined, further improvements in the age of initial diagnosis are suggested. The field study, data examined for children receiving treatment in the habilitation centers after the first three years post-implementation indicated that 53% underwent follow up testing by age three months. This figure was compared with the data collected in the telephone interview on compliance and satisfaction for parents of infants who had a reported failed screen for the NHSP in the years 2014–2015. The data from the interview indicated that 81% of infants were seen by three months. Although the improvement is commendable, it has not yet reached the target established by the NIH where all infants referred from a screen receive follow up by age three months.

While not directly examined, further improvements in the age of initial diagnosis are suggested from the comparison of the field data study covering the first three years post implementation and our telephone survey conducted five years post implementation. Data collected from charts of children enrolled in the habilitation centers indicated that in the first three years post implementation 53% underwent follow up testing by age three months. Our telephone interview on parental satisfaction and compliance for 2014–2015, showed that the 81% of infants were seen by three months. Although the improvement is commendable, it has not yet reached the target established by the NIH where all infants referred from a screen receive follow up by age three months.

The telephone interview on parental satisfaction and compliance provided valuable feedback regarding the impact of the hospital staff’s communication on the parents regarding the screening results. There’s a higher likelihood that parents will bring their children in for follow up testing before three months of age if they understood when the hospital staff explained the screen results. Attention paid to the parents’ understanding of the importance of the screening program can aid in parents’ bringing in their children for follow up testing before three months of age.

Success of a screening program depends on the ability to ensure follow up for referred infants. The National Health Service (UK) newborn hearing screening program (NHSP) uses a centrally organized computerized information system that records screening results and follow up until diagnosis. Professional staff with access to this system are able to ensure that all referred infants are tracked. As documented in other studies [25–27] a successful program includes scheduling a prompt appointment for follow up testing as well as efficient coordination between screening and diagnostic services. For the 2012/13 birth cohort in the UK, the proportion of referred infants attending a follow up appointment was reported at 82.5% within four weeks of the referral to undergo an audiology assessment [28].

Effective teamwork and communication between screening and audiological teams enables the British program to be more effective.

Division between responsibility for screening and diagnosis in Israel makes this challenging. The need for efficient information transfer has been identified with out of the box solutions to permit scheduling of follow up appointments in health fund approved diagnostic centers. Higher rates of infants receiving diagnostic audiologic testing could be accomplished if there was a dedicated preventive service team responsible for referring infants with a failed screen to be tested within a mandated maximum wait time of no more than three months of age. Since most parents brought their children for follow-up testing, the time delay documented can at least partially be explained by insufficient availability of testing facilities.

In the telephone survey where compliance and parental satisfaction were assessed, 83% of parents reported following up their referral with testing. This is comparable to what is reported in the US (71.3%) [8] and the UK (82.5%) [28]since their data refers to the time period up to three months after initial screening.

Issues identified which impact on compliance and experience with the program include parental anxiety, communication of screening results and assistance in scheduling follow-up. Another concern revealed in the parental interview was possible confusion regarding the screen results where parents reported their infants passed the screen (*n* = 42) although the sample was chosen based on hospital records indicating a failed test. Several of these parents (*n* = 12) also reported on follow up test results which wouldn’t have been necessary had the infants passed the initial screen.

A certain amount of anxiety appears to motivate families to follow up in a timely manner [29]. This concurs with our findings that parents showing heightened concern were more apt to follow through with the referral. We are interested in finding better ways to encourage compliance in this sensitive period of adjustment. Health provider communication is a key factor in decreasing the emotional impact of learning the hearing screening result. Staff communicating to parents failed screen results should be well informed, honest; offering a personalized explanation, avoid jargon, listen carefully, encourage questions, and recognize parental distress [30]. Still, much can be learned from the s direct assignment of audiology appointments by screeners at the point of screen completion that make the British program effective. Anxiety can also be reduced when the parents consult with their pediatrician or MCHC nurse who provide information and support. In all Israeli maternity hospitals, audiologists and speech and language pathologists manage the screening programs and their expertise in communicating with the parents is needed in the community where audiologic testing is performed.

### Limitations

For the data collected from the habilitation centers on the impact of the program from the first three years following implementation, the absence of a standard intake form hindered efforts to perform a thorough evaluation. The study included only children receiving treatment in habilitation centers and not necessarily all infants and children diagnosed with hearing impairment. These centers serve mostly children receiving disability benefits from the National Social Security. Our estimates on the impact of the program do not include children with less severe hearing impairment and those whose parents chose private treatment. Therefore, children with unilateral or minor hearing impairment were underrepresented. Thus, our estimates on prevalence of hearing impairment based on enrolled children less than one year of age are lower than the 1% threshold [1] which has been used to assess adequacy of screening.

We are unable to determine the yield of bilateral permanent childhood hearing loss from the screen for children in Israel as hearing loss is not yet included in the list of diseases or conditions requiring mandatory reporting.

While we acknowledge that the pre- and post-implementation outcome study design is flawed by the longer follow-up times for the pre-implementation group, the use of median age for assessing the targets, helps to mitigate this bias.

The telephone survey measuring parental compliance and satisfaction is a qualitative study on a relatively small sample of the larger population. This raises a concern regarding reliability of the data. Results cannot necessarily be generalized to all parents or even to a specific group of parents. Discrepancies among answers to certain questions in the survey were noted and it is unclear whether the confusion occurred either at the time of the interview or when the information was originally relayed.

With the data collected from the field research in the habilitation centers and from the telephone interviews, measures were reported on age at diagnosis and initiation of habilitation for two points in time. Further monitoring is necessary and will be repeated in the near future to assess program objectives and performance measurements.

## Conclusions

While instituting a national newborn hearing screening program in Israel has shown significant improvements in referring infants for diagnosis at a much earlier age, and enabling earlier initiation of habilitation for those who are in need of services, there are several aspects lacking in the program as compared to those in the UK, Australia and the US.

For Israel, there is no formal evaluation management protocol in place. The research described in this paper is the first attempt to quantify the progress of the national program. There are numerous features in the Israeli NHSP which are essential for recording and measuring the impact of the program, yet without the appropriately designated pathways for management and administration of evaluation, the invaluable data available and its transfer between the various care providers will be untapped and lost.

At present there is no real-time information transfer to the responsible health fund for newborns needing follow up testing or diagnosis. The division of responsibility for screening (hospitals) and diagnostic testing and habilitation (health funds) poses an additional barrier to monitoring program implementation. Infants who fail the screening test in the hospital are either referred for a repeat screen or a diagnostic exam at the health fund. The facilities for diagnostic testing need scaling up, i.e. establishing a protocol to indicate when re-screening is advised as opposed to diagnostic testing in order to reduce wait times that impair the efficacy of the program.

The recently updated directive from the Ministry of Health requires individual level reporting of screen results from hospitals into the computerized database. Once developed and implemented, this will enable the transfer of individual level information to the health funds to identify cases needing follow up and to generate reports for the MOH to periodically assess coverage, referral rates, need for services, etc. We also expect to include hearing loss under the age of five as a reportable condition on the mandatory listing of congenital malformations. Therefore, the cases identified can be tracked to ensure they receive the necessary services, diagnostic testing and treatment at habilitation centers. Hopefully the current number of cases with late diagnoses will be greatly decreased. In addition, further measures need to be investigated for reducing the wait time for follow-up testing to meet the objectives of Israel’s hearing screening program. Plans for a repeat assessment of the NHSP are currently underway with the intention of establishing a routine monitoring system.

## Abbreviations

A-ABR: Automated Auditory Brainstem Response
ANSD: Auditory Neuropathy Spectrum Disorders
MCHC: Mother and Child Health Clinic
MOH: Ministry of Health
NHSP: Newborn Hearing Screening Program
NIH: National Institute of Health
NSW SWISH: New South Wales Statewide Infant Screening Hearing Evaluation
OAE: Otoacoustic Emissions
PHI: Permanent Hearing Impairment
RR: Referral Rate
UK: United Kingdom
US: United States

## Footnotes

1 Findings from the Household Expenditure Survey 2012 provide data on the Israeli households’ ownership and expenditure on communication and entertainment products which states that 95% of Israeli households have at least one mobile phone (https://www.cbs.gov.il/en/mediarelease/Pages/2013/Findings-from-the-2012-Household-Expenditure-Survey-on-the-Housing-in-Israel.aspx Media Release 343/2013, December 11, 2013)
